# Reply to Paterson, R.R.M. Comment on “Castillo et al. *Ganoderma zonatum* Is the Causal Agent of Basal Stem Rot in Oil Palm in Colombia. *J. Fungi* 2022, *8*, 230”

**DOI:** 10.3390/jof8090945

**Published:** 2022-09-08

**Authors:** Sandra Yulieth Castillo, María Camila Rodríguez, Luis Felipe González, León Franky Zúñiga, Yuri Adriana Mestizo, Héctor Camilo Medina, Carmenza Montoya, Anuar Morales, Hernán Mauricio Romero, Greicy Andrea Sarria

**Affiliations:** 1 Pest and Disease Program, Colombian Oil Palm Research Center—Cenipalma, Bogotá 111211, Colombia; 2 Biology and Plant Breeding Program, Colombian Oil Palm Research Center—Cenipalma, Bogotá 111211, Colombia; 3 Department of Biology, National University of Colombia, Bogotá 111321, Colombia; 4 Cenipalma, Experimental Field Palmar de La Vizcaína, Km 132 Vía Puerto Araujo-La Lizama, Santander, Barrancabermeja 111611, Colombia

Reader Q: There was no morphological information provided for the isolated specimens, and so it is not known if they are even Ganoderma [1]:

Editors answer: 

Since this is not a new mushroom species, it is not necessary to use morphological characteristics, and phylogeny has supported the identification.

Authors answer:

Currently, in Colombia, there is 590,188 ha planted with oil palm, distributed geographically in over four zones: Eastern (274,596 ha), North (111,781 ha), Central (180,928 ha), and Southwest (22,883 ha). Basal stem rot is restricted to only two plantations in the North Zone, with 2000 and 886 ha, respectively, for each plantation, and cumulative incidences of 3% in periodic evaluations since 2015. In contrast, Roslan and Idris [2] reported more than 400 thousand ha of palm oil affected by Basal Stem Rot in Malaysia and Indonesia. 

In Colombia, the phytopathology team of Cenipalma has been working since 2008 to identify the causal agent of Basal Stem Rot, through the recognition of symptoms and isolation of associated microorganisms, from both infected tissue and basidiocarps. Based on the morphological characteristics of 12 basidiocarps and different taxonomic keys [3,4,5], the team concluded that they belong to the genus Ganoderma [6]. Several works also aimed to standardize the inoculation methods with these isolates, using different substrates and hosts. In 2019 the inoculation methodology was implemented using rubber wood blocks colonized with Ganoderma, finding pathogenicity in nursery plants and recovering the microorganism from the affected plants, thus confirming Koch’s postulates. These results are part of this article. The results of our study are part of the first phase of research, where the identity of Ganoderma isolates associated with Basal Stem Rot in Colombia is confirmed through pathogenicity tests and molecular identification. We are currently working on increasing the number of isolates from the only two plantations in which this disease is present in Colombia to carry out genetic and biological diversity studies to complement the information published in this article. 

Considering the above, and as the main objective of the article was not to carry out the taxonomy of the fungus, since it does not correspond to a new species associated with this disease, the phylogeny was carried out using three genetic markers for its identification (ITS, TEF, RPB ), which have been reported by Xing et al. [7], Hapuarachchi et al. [8], Cabarroi-Hernández et al. [9], Liu et al. [10] in studies of genetic diversity of Ganoderma [10].

Reader Q: Assuming they are, *Ganoderma miniatocinctum* was not included in the molecular analysis and yet is one of the pathogens of oil palm [1].

Editors answer: 

The paper’s main aim is not the mushroom’s taxonomy; the authors have conducted phylogeny using three gene markers for the identification. 

Author answer: 

*Ganoderma miniatocinctum* sequences were not used because the sequences in the present study did not show identity with *G. miniatocinctum* database sequences. Additionally, three markers were used for the investigation to identify the species (*G. zonatum*) of the isolates associated with Basal Stem Rot in Colombia. The number of isolates that were evaluated was restricted to a single Colombia palm area. Likewise, as mentioned in the article, positive pathogenicity results were obtained in addition to the phylogenetic study, which corroborated the conclusions. Potentially, in the future, and as mentioned in the paper’s perspective, when there are more isolations, a taxonomic and genetic diversity study will be proposed to show whether there is a diversity of Ganoderma species associated with BSR in Colombia [11].
